# Supplementary material for: Control of epithelial tissue organization by mRNA localization
Source: Nat Commun. 2025 Jun 5;16:5216. doi: 10.1038/s41467-025-60532-8 (PMC12141530; doi:10.1038/s41467-025-60532-8)
Supplement: Supplementary file 1 — Supplementary Information [file 41467_2025_60532_MOESM1_ESM.pdf]

Supplementary Information

Control of Epithelial Tissue Organization by mRNA Localization

Devon E. Mason, Thomas D. Madsen, Alexander N. Gasparski, Dong Kong, Neal Jiwnani, Terry Lechler, Jadranka Loncarek, Roberto Weigert, Ramiro Iglesias-Bartolome, Stavroula Mili

Supplementary Figures 1-9
Supplementary Tables 1-3

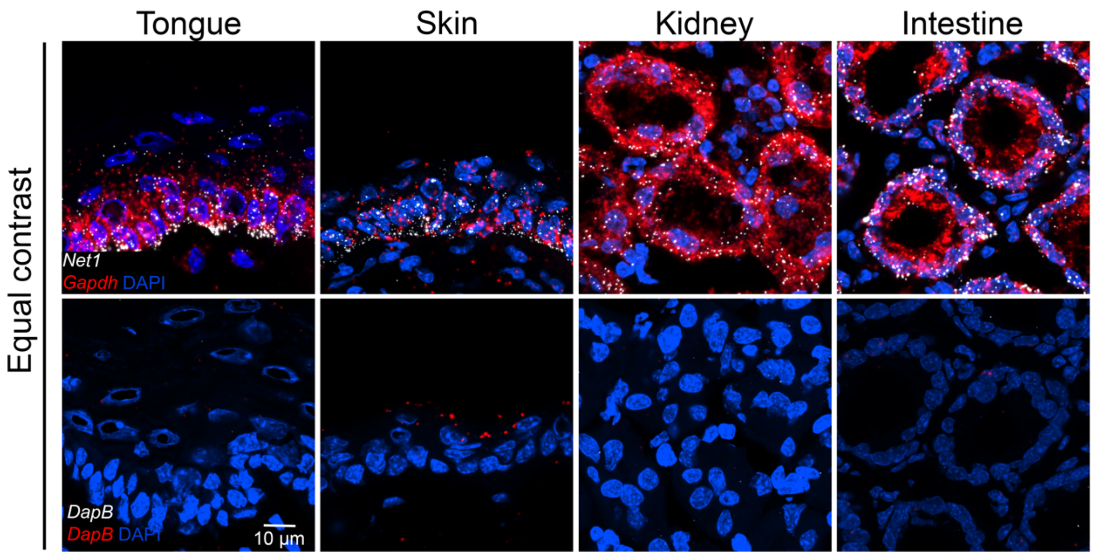

Supplementary Fig. 1: mRNA-specific probes label endogenous mRNAs in mouse tissue. Representative images of *Net1* and *Gapdh* mRNA in sections of mouse tongue, skin, kidney, and intestine. The degree of non-specific signal was qualitatively assessed using the bacterial dihydrodipicolinate reductase (*DapB*) mRNA as a negative control.

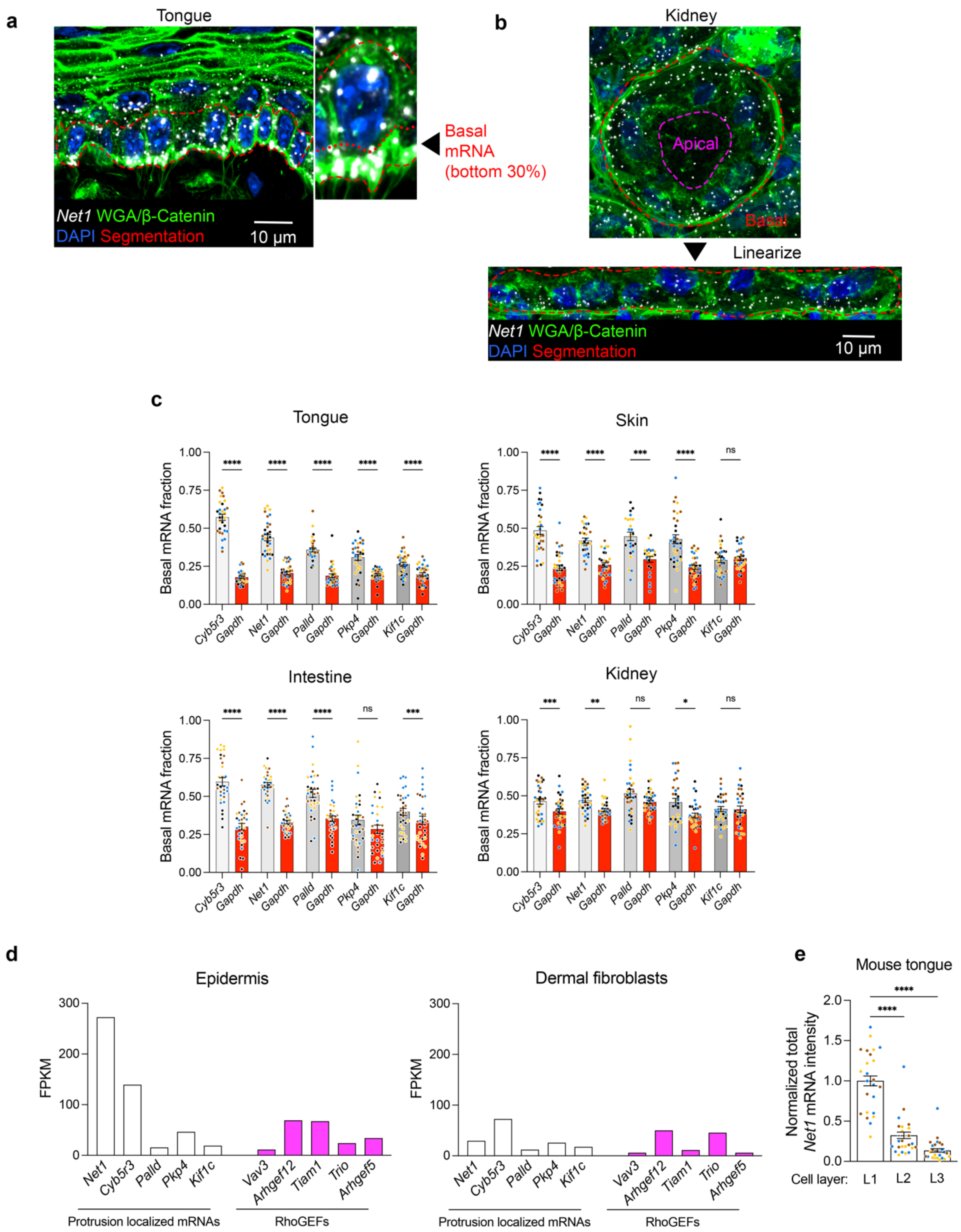

Supplementary Fig. 2: Protrusion localized mRNAs are basally localized across architecturally distinct tissues. a, Representative image of mouse tongue tissue stained for *Net1* mRNA and a combination of WGA and β-catenin. Manual segmentation of the BM-attached

epithelial cell layer is indicated by a red dashed line. mRNA accumulation in the bottom 30%, referred to as the basal mRNA fraction, is visualized in the inset. **b**, Representative tubular epithelial sheet from the kidney stained for *Net1* mRNA and a combination of WGA and β-catenin. For analysis, the image was first linearized and subsequently analyzed using the approach taken in stratified tissues. **c**, Basal mRNA fraction of several protrusion-localized mRNAs (*Cyb5r3*, *Net1*, *Palld*, *Pkp4*, and *Kif1c*; grey bars) and of the *Gapdh* control mRNA (red bars) in mouse tongue, skin, intestine, and kidney. **d**, Protrusion-localized and RhoGEF mRNA expression in the skin epidermis vs. dermal fibroblasts derived from published datasets. *Net1* expression is notably higher in the epidermis to the point where it qualifies as a signature gene for the basal keratinocytes of the skin. **e,** Relative fluorescent intensity of *Net1* mRNA in tongue keratinocytes from the 1st (BM-attached), 2nd, and 3rd epithelial layers. Bar graph data are measurements from individual ROIs with mean ± SEM; n = 27-32 ROIs from N = 3-4 mice. Data from individual mice are represented with different colors. * $P < 0.05$, ** $P < 0.01$, *** $P < 0.001$, **** $P < 0.0001$ by repeated measures ANOVA followed by Sidak's multiple comparisons test (c) or Kruskal-Wallis ANOVA followed by Dunn's multiple comparisons test (e). Source data are provided as a Source Data file.

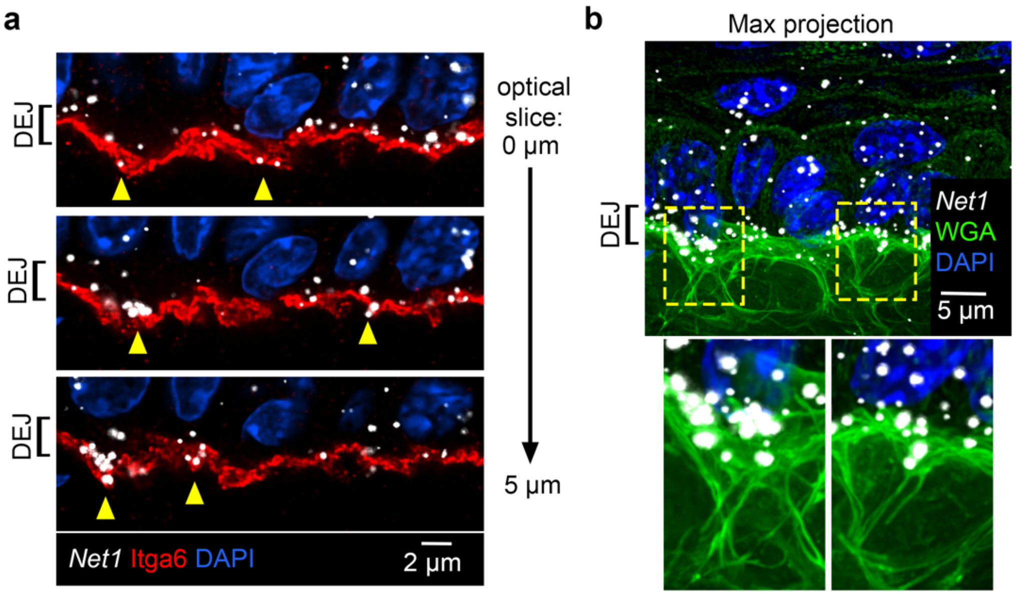

Supplementary Fig. 3: Basal keratinocytes of the mouse tail skin have protrusion-like structures connected to WGA positive dermal fibers at the DEJ . a, Representative images of mouse tail skin showing *Net1* mRNA at the DEJ. The basal keratinocyte plasma membrane is visualized by Itga6 in sequential optical slices and exhibits protrusion-like structures. Yellow arrowheads indicate *Net1* mRNA at protrusion-like structures. **b**, *Net1* mRNA and WGA staining of mouse tail skin. Bottom panels: zoomed in regions, indicated by yellow boxes, highlighting WGA-positive dermal fibers connecting with the basal keratinocyte cell membrane. Image is a max-intensity projection of ~5 um in the z-axis.

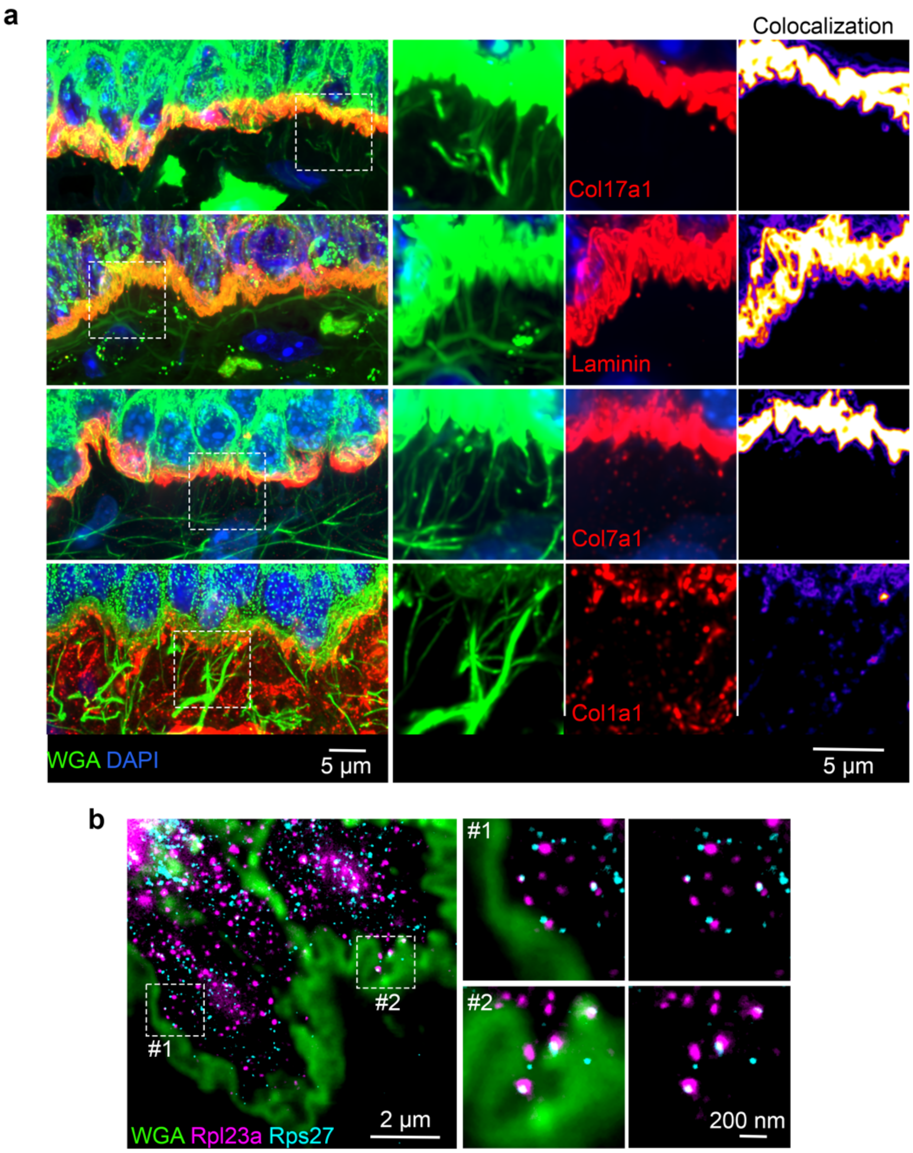

Supplementary Fig. 4: DEJ fibers are not comprised of hemidesmosome, basement membrane, or anchoring components. **a,** Representative images of mouse tongue tissue stained with WGA as well as with a panel of DEJ antibodies against Col17a1, pan-laminin, Col7a1, or Col1a1. Left panels: channel overlay images. Middle panels: individual channels (WGA:green; DEJ components: red) for the magnified boxed regions. Colocalization panels show pixel intensity overlap between WGA and DEJ channels. There was modest co-localization between DEJ fibers and Col1a1. Other DEJ components overlap with WGA at the region of the plasma membrane but not with WGA-positive fibers below the DEJ. **b,** STED super-resolution imaging of mouse tongue tissue stained for WGA, Rpl23a and Rps27. Area shown focuses on protrusion-like structures of BM-attached keratinocytes. Boxed regions are magnified in the right panels to show

colocalization, or proximity, of Rpl23a and Rps27 signals, consistent with their presence in translating 80S ribosomes.

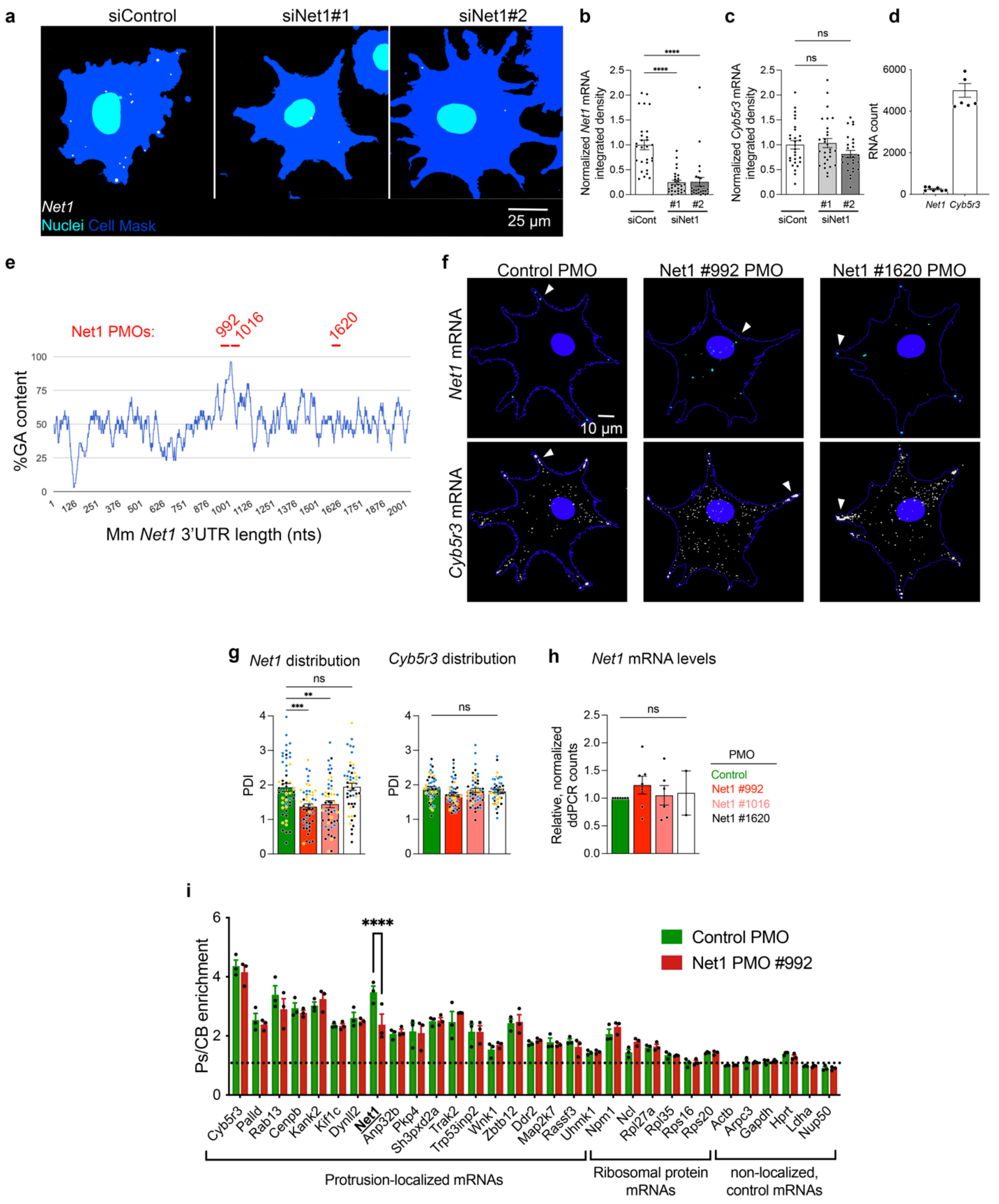

Supplementary Fig. 5: PMOs targeting the Mm *Net1* GA-rich region specifically alter *Net1* mRNA localization. **a**, Representative images of *Net1* mRNA detected using mRNA-specific FISH probes in NIH/3T3 fibroblasts after transfection with either non-targeting control or *Net1* siRNA. **b**, **c,** *Net1* (b), but not *Cyb5r3* (c), mRNA signal is significantly reduced in cells treated

with *Net1* siRNA demonstrating the specificity of the signal. n = 26-28 cells. **d,** NanoString nCounter RNA counts of *Cyb5r3* and *Net1* mRNAs from total NIH/3T3 cell lysate. *Cyb5r3* mRNA is ~20 times more abundant than *Net1* in agreement with the relative signals obtained by FISH (see panel f for representative images). **e,** GA content in 30nt windows along the *Net1* 3'UTR. Experimental Net1 PMOs were designed to target the *Net1* GA-rich region (#992 and #1016) or a different region (#1620) as a control (red lines indicate PMO locations). **f**, Representative images of *Net1* and *Cyb5r3* mRNA distribution in NIH/3T3 fibroblasts transfected with Control or Net1 targeting PMOs. Cell boundaries are indicated by a blue line. Arrowheads point to areas of RNA accumulation. Note that Net1 #992 PMO leads to perinuclear *Net1*. **g**, *Net1* and *Cyb5r3* mRNA distribution after PMO transfection as measured by a peripheral distribution index (PDI). PDI is an intensity weighted measure of the distribution of an RNA population relative to the center of the nucleus. A value above 1 is more peripheral, 1 is diffuse, and a value below 1 is a more perinuclear mRNA. n = 51 cells in 3 independent biological replicates. Data from independent experiments are shown in different colors. **h**, ddPCR count of *Net1* mRNA normalized to a housekeeping gene. n=2-7 biological and technical replicates **i**, Protrusion (Ps) and cell body (CB) fractions were isolated from 3T3 cells extending protrusions through transwell pores. The indicated mRNAs were detected by nanoString analysis to calculate a Ps/CB enrichment ratio. n=3. Bar graph data are individual measurements with mean ± SEM. ** $P < 0.01$, *** $P < 0.001$, **** $P < 0.0001$, ns: non-significant by ordinary one-way ANOVA followed by Dunnett's multiple comparison test (b, c, g), mixed effects analysis with Dunnett's multiple comparison test (h), or 2-way ANOVA followed by Sidak's multiple comparison test (i). Source data are provided as a Source Data file.

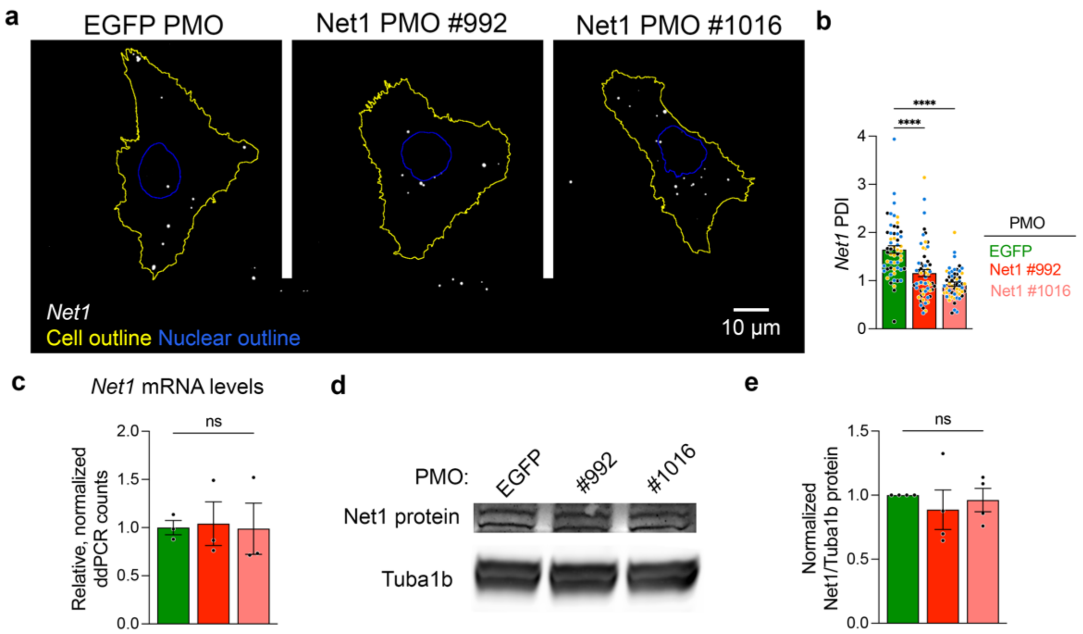

Supplementary Fig. 6: *Net1* PMOs alter *Net1* mRNA localization without affecting mRNA or protein abundance in mouse keratinocytes in vitro. a, Representative images of *Net1* mRNA localization in immortalized mouse keratinocytes treated with PMOs targeting *EGFP* or *Net1* mRNAs. **b**, Peripheral distribution of *Net1* measured by PDI in PMO treated cells. n = 56 cells in 3 independent biological replicates. Data from independent experiments are shown in different colors. **c**, ddPCR count of *Net1* mRNA normalized to a housekeeping gene *GusB*. n=3. **d**, Representative images of western blot detecting Net1 and Tuba1b protein from whole keratinocyte lysates. **e**, Quantification of Net1 protein normalized to Tuba1b. n=4. Bar graph data are individual measurements with mean ± SEM. **** $P < 0.0001$, ns: non-significant by Kruskal-Wallis ANOVA followed by Dunn's multiple comparison test (b), or ordinary one-way ANOVA followed by Dunnett's multiple comparison test (c, e). Source data are provided as a Source Data file.

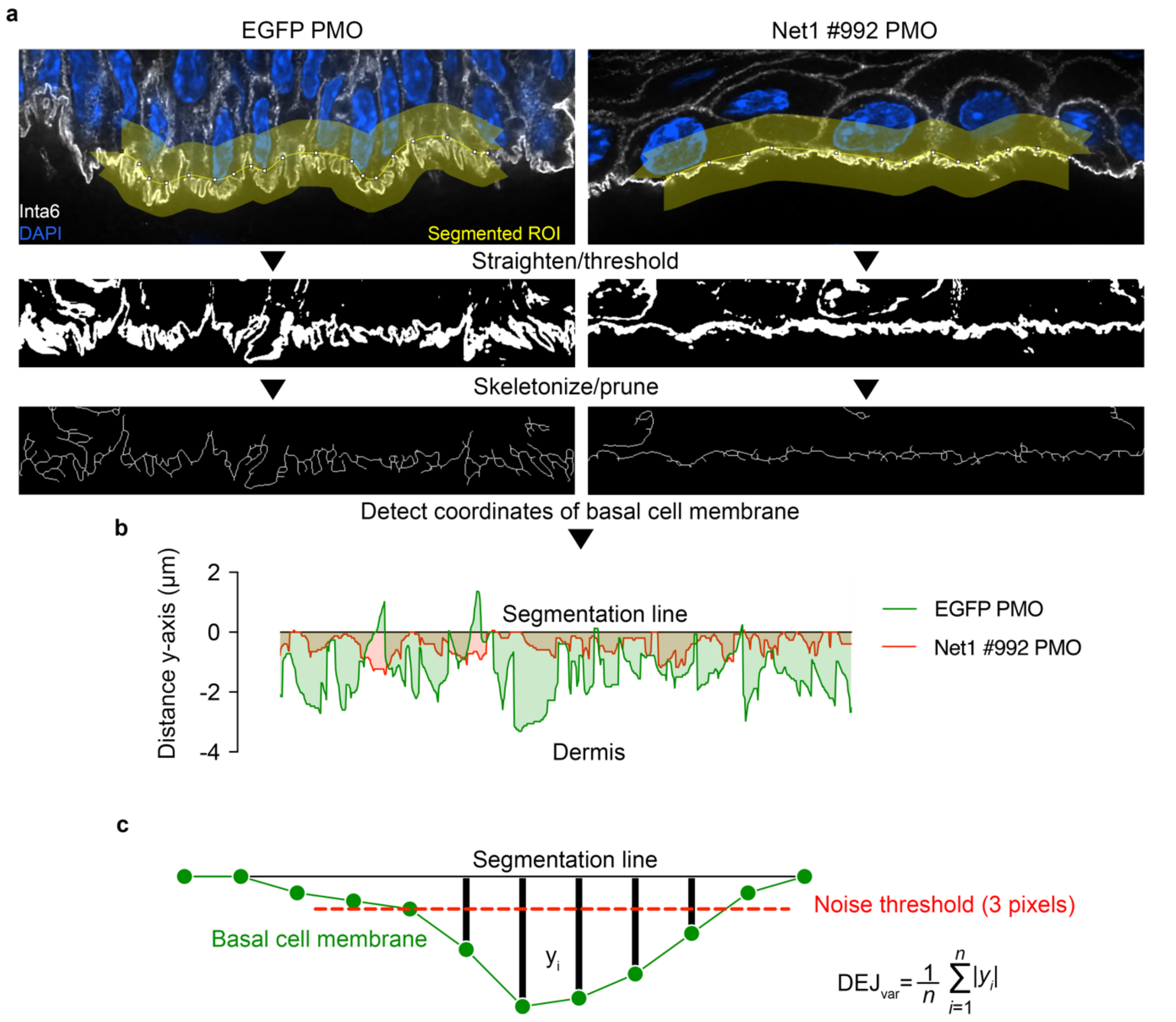

Supplementary Fig. 7: Analysis pipeline for measuring membrane topographical variation in the DEJ. a, For analysis, individual optical slices from tissue sections stained for Itga6 were used. Regions of the DEJ were isolated by drawing a segmented line ROI (120 pixel (7.8 µm) wide) centered on the basal cell membrane. The upper limits of protrusion-like structures were used as a reference. The ROI was digitally straightened and thresholded followed by skeletonization and pruning of incomplete membrane segments. **b**, From the mask in (a) the most basal coordinates in each x-position were derived. These basal coordinates were used to estimate y-axis variation relative to the original segmentation line. The graphical representation depicts x-position coordinates for the representative images from EGFP or Net1 PMO treated tissue. **c**, DEJ variation (DEJ_{var}) was calculated as the average distance between the basal cell membrane coordinates (described in b) and the segmentation line. To limit the degree to which segmentation variability can influence the result a noise threshold of 3 pixels (~.2 µm) was applied so that

vertical displacement less than 3 pixels were set to 0. Multiple optical slices and segmentation lines were used to get a variability estimate across at least 100 µm of the DEJ for a single ROI, at least 5 ROIs were measured per animal. Source data are provided as a Source Data file.

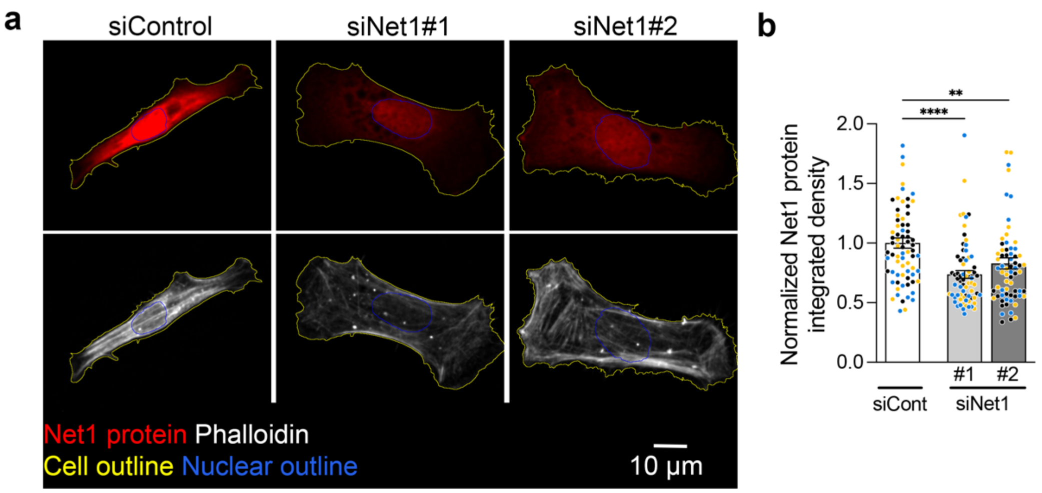

Supplementary Fig. 8: Net1 antibody validation for immunofluorescence in mouse keratinocytes. **a**, Representative Net1 protein immunofluorescence counterstained with phalloidin to visualize the actin cytoskeleton. Cells were treated with either a control or two different Net1 targeting siRNAs. Note: Net1 protein is predominantly nuclear in many in vitro cultured cells. **b**, Relative immunofluorescence intensity of Net1 protein. n = 22-28 cells per condition from 3 independent experiments. ** $P < 0.01$, **** $P < 0.0001$ by Kruskal-Wallis ANOVA followed by Dunn's multiple comparison test. Source data are provided as a Source Data file.

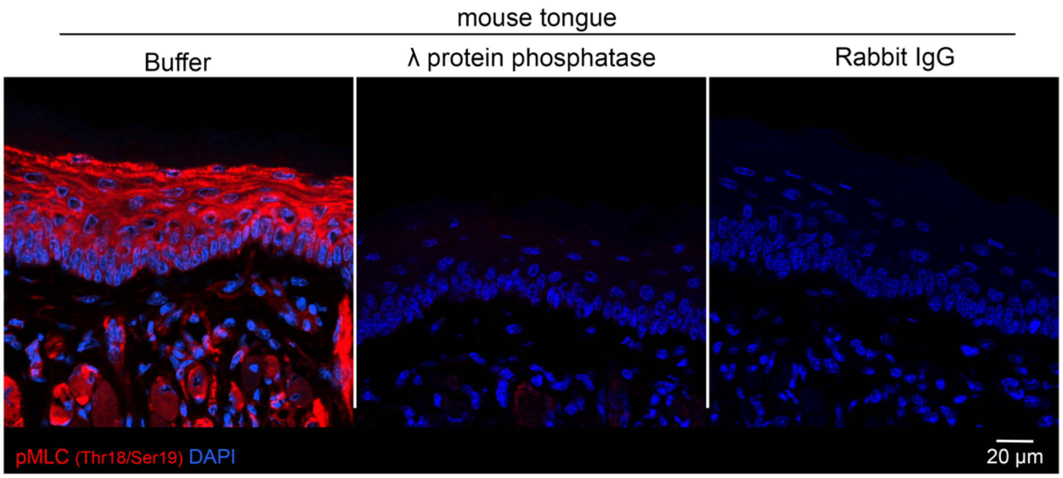

Supplementary Fig. 9: Myosin light chain (Thr18/Ser19) phosphorylation can be detected in vivo by immunofluorescence. Fixed mouse tongue sections were treated with λ protein phosphatase or buffer then stained with pMLC (Thr18/Ser19) primary antibody or Rabbit IgG. Immunofluorescent detection using an anti-rabbit secondary antibody indicated that pMLC primary antibody binding is diminished in tissues pre-treated with a serine, threonine, and tyrosine phosphatase.

Supplementary Table 1: PMO and siRNA sequences used in this work

**PMO target**	**Sequence (5'-3')**
Mm-*Net1*-992	TCCCTTCCCTATCCCAGACTCCTG
Mm-*Net1*-1016	AGCTACTCTTCTCTTCTCTTCCCT
Mm-*Net1*-1619	ACTAAACTATGGACTTTTCTTATCA
EGFP	ACAGCTCCTCGCCCTTGCTCACCAT
Scrambled (*Net1*-992)	ACTTGCCTACGCCTCCTCTCTACC

**siRNA target**	**Target sequence (5'-3')**
Mm-*Net1* (siRNA #1)	AAGGGTTACTCTGTACCAGAA
Mm-*Net1* (siRNA #2)	CAGAGGGAGCATGGAATTGTA

Supplementary Table 2: Antibodies used in this work

**Antibody**	**Source**	**Product number**	**Concentration for IF**	**Validation**
Rabbit anti-β-catenin	Sigma	HPA029159	1:200	Atlas antibody validated for IHC and ICC-IF
Rabbit anti-PCNA	Cell signaling technology	13110S	1:400 (Methanol permeabilization)	https://www.cellsignal.com/products/primary-antibodies/pcna-d3h8p-xp-rabbit-mab/13110?srsltid=AfmBOoqtipKPPcDYl8DVtFtGt9gwEqi1VHXsYeQmwZVsayAzdgicO-U8
Chicken anti-Krt5	Biolegend	905901	1:800	https://www.biolegend.com/fr-ch/products/keratin-5-polyclonal-chicken-antibody-purified-10957?GroupID=GROUP26
Rabbit anti-Krt13	Abcam	92551	1:800	https://www.abcam.com/en-us/products/primary-antibodies/cytokeratin-13-antibody-epr3671-ab92551?srsltid=AfmBOorX57mJcFLwLHwZ3_8L0eauwi0mK5CYo7Uj01fn9wICrbYttTD-
Rat anti-Itga6	Biolegend	313602	1:800	https://www.biolegend.com/de-at/products/purified-anti-human-mouse-cd49f-antibody-2604
Rabbit anti-Fbn2	Novus Biologicals	NBP1-88169	1:200	https://www.novusbio.com/products/fibrillin-2-antibody_nbp1-88169?srsltid=AfmBOooEliFlqdY1dDZv_LPZE8arGVSSoV0NnpkztX6vliyKtauOC77J
Rabbit anti-Net1 protein	Bethyl	A303-138A	1:200	This work (supplementary figure S8) and Gasparski et al (fig S12b, DOI: 10.1016/j.molcel.2023.06.036)
Rabbit anti-pMLC (Thr18/Ser19)	Cell signaling technology	95777	1:200	This work (Supplementary figure S9)
Rat anti-HA	Roche	11867423001	1:100	This work Figure 5 and Ning et al 2021 (DOI: 10.1016/j.stem.2020.11.002)
Rabbit anti-Col17a1	Abcam	ab184996	1:200	https://www.abcam.com/en-us/products/primary-antibodies/collagen-xvii-antibody-epr18614-ab184996?srsltid=AfmBOorz__H5_-RjW-78ChutrgDVH67FpqsPkg_TWwe-YVKNHvN97Il0#
Rabbit anti-Laminin	Invitrogen	PA5-22901	1:100	https://www.thermofisher.com/antibody/product/Laminin-Antibody-Polyclonal/PA5-22901
Rabbit anti-Col7a1	Novus	NBP2-37900	1:100	https://www.novusbio.com/products/collagen-vii-antibody_nbp2-37900?srsltid=AfmBOoqoJ8h8oIIbZOdx2FG_-evKlvPHqdxT8AbRdFVOABoaghIFaHME
Rabbit anti-Col1a1	Abcam	ab34710	1:100 (use on unfixed fresh frozen tissue)	https://www.abcam.com/en-us/products/primary-antibodies/collagen-i-collagen-iii-antibody-ab34710?srsltid=AfmBOoolBu1ShhOjYUSDWHn6uHkT_kBFPXzUbJJJgqyTSwMCIWPGl1Ta
Goat anti-Rps27	GeneTex	GTX24385	1:100	https://www.genetex.com/Product/Detail/MPS1-antibody/GTX24385?srsltid=AfmBOorLQTwGi0epjwa8t3Yr5QPc11vwgFpSg9lQKbeb3uH6fX8U7e_s
Rabbit anti-Rpl23a	Abcam	ab157110	1:300	https://www.abcam.com/en-us/products/primary-antibodies/rpl23a-antibody-ab157110?srsltid=AfmBOopd-f9wo7r8pv4OEyhFjr5LYZYlzqQO3qAqJi0mPHuOuZvw_4Kr

Supplementary Table 3: Probes used for RNA visualization

Probe (species/target mRNA)	Source	Product number
Mm-*Net1*	RNAscope probes (ACD)	1080571-C1
Mm-*Cyb5r3*		1200561-C2
Mm-*Pkp4*		1200551-C2
Mm-*Palld*		822611-C2
Mm-*Kif1c*		822461-C2
Mm-*Gapdh*		442871-C3
Hu-*NET1*		810461-C1
Hu-*GAPDH*		442201-C2
negative control		320871
Net1 PMO 1016 detection probe		1258881-S1
Mm-*Net1*-Type 1	Affymetrix (Thermo Fisher Scientific)	VA1-20646
Mm-*Cyb5r3*-Type 6		VA6-3171469
