# Supplementary material for: Control of epithelial tissue organization by mRNA localization
Source: Nat Commun. 2025 Jun 5;16:5216. doi: 10.1038/s41467-025-60532-8 (PMC12141530; doi:10.1038/s41467-025-60532-8)
Supplement: Supplementary file 3 — Description of Additional Supplementary Files [file 41467_2025_60532_MOESM3_ESM.pdf]

Description of additional Supplementary files

File name: Supplementary Movie 1

Description: *Net1* mRNA and DEJ component staining in mouse tongue. Representative serial optical sections of mouse tongue. Upper panels: The basal cell membrane is visualized with the basal cell membrane marker Itga6, with or without overlay with *Net1* mRNA signal. Bottom panels: Visualization of the basal cell membrane and dermal fibers through WGA staining, with or without overlay with *Net1* mRNA signal. ~3 μm thick serial optical sections were acquired with a ~0.065 μm step size.

File name: Supplementary Movie 2

Description: *Net1* mRNA and DEJ component staining in mouse tongue. An additional representative serial optical sectioning of mouse tongue. Upper panels: The basal cell membrane is visualized with the basal cell membrane marker Itga6, with or without overlay with *Net1* mRNA signal. Bottom panels: Visualization of the basal cell membrane and dermal fibers through WGA staining, with or without overlay with *Net1* mRNA signal. ~3 μm thick serial optical sections were acquired with a ~0.065 μm step size.

File name: Supplementary Movie 3

Description: *Net1* mRNA distribution influences epithelial cell interaction with the DEJ. Serial optical slices of mouse tongues treated with EGFP, Scrambled, Net1 #992, and Net1 #1016 PMOs. Regions shown are displayed in Figure 3j. The basal cell membrane is visualized with Itga6 (grey) and nuclei are stained with DAPI. ~6 μm thick serial optical sections were acquired with a 0.1 μm step size.

File name: Supplementary Code 1

Description: Matlab script used for measuring mRNA signal intensity across a cell layer

File name: Supplementary Code 2

Description: Fiji macro used to measure Net1 protein distribution

File name: Supplementary Code 3

Description: Fiji macro used to measure DEJ variation
