# Supplementary material for: Control of epithelial tissue organization by mRNA localization
Source: Nat Commun. 2025 Jun 5;16:5216. doi: 10.1038/s41467-025-60532-8 (PMC12141530; doi:10.1038/s41467-025-60532-8)
Supplement: Supplementary file 10 — Reporting Summary [file 41467_2025_60532_MOESM10_ESM.pdf]

nature portfolio

Corresponding author(s): Dr. Stavroula Mili
Dr. Devon Mason

Last updated by author(s): Mar 31, 2025

Reporting Summary

Nature Portfolio wishes to improve the reproducibility of the work that we publish. This form provides structure for consistency and transparency in reporting. For further information on Nature Portfolio policies, see our Editorial Policies and the Editorial Policy Checklist.

Statistics

For all statistical analyses, confirm that the following items are present in the figure legend, table legend, main text, or Methods section.

n/a	Confirmed	
☐	☒	The exact sample size (n) for each experimental group/condition, given as a discrete number and unit of measurement
☐	☒	A statement on whether measurements were taken from distinct samples or whether the same sample was measured repeatedly
☐	☒	The statistical test(s) used AND whether they are one- or two-sided<br>*Only common tests should be described solely by name; describe more complex techniques in the Methods section.*
☐	☒	A description of all covariates tested
☐	☒	A description of any assumptions or corrections, such as tests of normality and adjustment for multiple comparisons
☐	☒	A full description of the statistical parameters including central tendency (e.g. means) or other basic estimates (e.g. regression coefficient) AND variation (e.g. standard deviation) or associated estimates of uncertainty (e.g. confidence intervals)
☐	☒	For null hypothesis testing, the test statistic (e.g. F, t, r) with confidence intervals, effect sizes, degrees of freedom and P value noted<br>*Give P values as exact values whenever suitable.*
☒	☐	For Bayesian analysis, information on the choice of priors and Markov chain Monte Carlo settings
☒	☐	For hierarchical and complex designs, identification of the appropriate level for tests and full reporting of outcomes
☒	☐	Estimates of effect sizes (e.g. Cohen's d, Pearson's r), indicating how they were calculated

Our web collection on statistics for biologists contains articles on many of the points above.

Software and code

Policy information about availability of computer code

Data collection	Imaging data were collected and processed using Nikon elements (5.21.03, Build 1489), Carl Zeiss Zen 2.3 or STEDYCON browser-based control software (Abberior Instruments)
Data analysis	Imaging data were analyzed using Matlab (R2021b-R2023b), Fiji(2.16.0/1.54g), Imaris (v9.9.0) or HALO image analysis platform (Indica Labs)

For manuscripts utilizing custom algorithms or software that are central to the research but not yet described in published literature, software must be made available to editors and reviewers. We strongly encourage code deposition in a community repository (e.g. GitHub). See the Nature Portfolio guidelines for submitting code & software for further information.

Data

Policy information about availability of data

All manuscripts must include a data availability statement. This statement should provide the following information, where applicable:

- Accession codes, unique identifiers, or web links for publicly available datasets
- A description of any restrictions on data availability
- For clinical datasets or third party data, please ensure that the statement adheres to our policy

Previously published sequencing data that were reanalyzed here are available under accession codes GSE129218 (https://www.ncbi.nlm.nih.gov/geo/query/acc.cgi?acc=GSE129218) and GSE77197 (https://www.ncbi.nlm.nih.gov/geo/query/acc.cgi?acc=GSE77197) in the NCBI Gene Expression Omnibus (GEO) database. All other data supporting the findings of this study are contained within the paper and its supplementary files. Source data are provided with this paper.

Research involving human participants, their data, or biological material

Policy information about studies with human participants or human data. See also policy information about sex, gender (identity/presentation), and sexual orientation and race, ethnicity and racism.

Reporting on sex and gender	All human patient derived samples have been de-identified in accordance with NIH standards. This includes irrevocable removal of all identifiable information including age, gender, sex, and race.
Reporting on race, ethnicity, or other socially relevant groupings	*Please specify the socially constructed or socially relevant categorization variable(s) used in your manuscript and explain why they were used. Please note that such variables should not be used as proxies for other socially constructed/relevant variables (for example, race or ethnicity should not be used as a proxy for socioeconomic status).* *Provide clear definitions of the relevant terms used, how they were provided (by the participants/respondents, the researchers, or third parties), and the method(s) used to classify people into the different categories (e.g. self-report, census or administrative data, social media data, etc.)* *Please provide details about how you controlled for confounding variables in your analyses.*
Population characteristics	*Describe the covariate-relevant population characteristics of the human research participants (e.g. age, genotypic information, past and current diagnosis and treatment categories). If you filled out the behavioural & social sciences study design questions and have nothing to add here, write "See above."*
Recruitment	*Describe how participants were recruited. Outline any potential self-selection bias or other biases that may be present and how these are likely to impact results.*
Ethics oversight	Under National Institutes of Health protocols, the use of biospecimens from de-identified discarded human tissue does not meet the regulatory criteria for human subject research and therefore institutional review board review or informed consent are waived.

Note that full information on the approval of the study protocol must also be provided in the manuscript.

Field-specific reporting

Please select the one below that is the best fit for your research. If you are not sure, read the appropriate sections before making your selection.

☒ Life sciences ☐ Behavioural & social sciences ☐ Ecological, evolutionary & environmental sciences

For a reference copy of the document with all sections, see nature.com/documents/nr-reporting-summary-flat.pdf

Life sciences study design

All studies must disclose on these points even when the disclosure is negative.

Sample size	Preliminary and published experiments were used to determine an appropriate sample size, including additional animals/experiments to account for sample loss.
Data exclusions	No animal/replicates were excluded from the final analysis included in this manuscript.
Replication	Replication was confirmed by performing experiments in multiple mice and across independent in vitro experiments (>3 replicates in every case).
Randomization	Animals were randomly assigned to one of the treatment groups.
Blinding	The investigators were not blinded to the experimental groups. Instead standardized procedures were applied evenly across groups.

Reporting for specific materials, systems and methods

We require information from authors about some types of materials, experimental systems and methods used in many studies. Here, indicate whether each material, system or method listed is relevant to your study. If you are not sure if a list item applies to your research, read the appropriate section before selecting a response.

Materials & experimental systems

n/a	Involved in the study	
	x	Antibodies
	x	Eukaryotic cell lines
x		Palaeontology and archaeology
	x	Animals and other organisms
x		Clinical data
x		Dual use research of concern
x		Plants

Methods

n/a	Involved in the study	
x		ChIP-seq
x		Flow cytometry
x		MRI-based neuroimaging

Antibodies

Antibodies used	A list of antibodies used in this study are included in Supplementary Table 2
Validation	All antibodies have been validated with respect to their specific application by the manufacturer or have been used extensively in the literature. We directly validated the specificity of antibodies targeting pMLC (Thr18/Ser19) and Net1 in Supplementary Figure 8 and 9.

Eukaryotic cell lines

Policy information about cell lines and Sex and Gender in Research

Cell line source(s)	NIH/3T3 mouse fibroblast cells were obtained from ATCC (cat# CRL-1658) . Mouse keratinocytes were isolated from tail skin of male mixed background mice (FVB/N and C57) and immortalized by infecting with lentiviruses expressing SV40 Large T Antigen.
Authentication	NIH/3T3 mouse fibroblast cells were obtained from ATCC and have not been authenticated. Immortalized mouse keratinocytes were validated by testing for expression of a panel of keratinocyte markers.
Mycoplasma contamination	Cell lines have tested negative for mycoplasma contamination.
Commonly misidentified lines (See ICLAC register)	No misidentified cell lines were used in this study

Animals and other research organisms

Policy information about studies involving animals; ARRIVE guidelines recommended for reporting animal research, and Sex and Gender in Research

Laboratory animals	C57BL/6/NCr mice and TRE-Arhgef11/Krt14-rtTA mice were used for each experiment. All mice were between 4-12 weeks old.
Wild animals	This study did not involve wild animals
Reporting on sex	An even distribution of Male and Female mice were used for essential experiments using PMOs. There where no sex-specific effects detected in several key metrics of tissue morphology suggesting PMOs have a comparable effect in males and females. As a result aggregate data is shown for each assay.
Field-collected samples	No samples were collected from the field
Ethics oversight	This study was performed under the ethical oversight of the Institutional Animal Care and Use Committees of the National Cancer Institute (National Institutes of Health, Bethesda, MD) and Duke University (Durham, NC; Protocol #: A255-23-12)

Note that full information on the approval of the study protocol must also be provided in the manuscript.

Plants

Seed stocks	*Report on the source of all seed stocks or other plant material used. If applicable, state the seed stock centre and catalogue number. If plant specimens were collected from the field, describe the collection location, date and sampling procedures.*
Novel plant genotypes	*Describe the methods by which all novel plant genotypes were produced. This includes those generated by transgenic approaches, gene editing, chemical/radiation-based mutagenesis and hybridization. For transgenic lines, describe the transformation method, the number of independent lines analyzed and the generation upon which experiments were performed. For gene-edited lines, describe the editor used, the endogenous sequence targeted for editing, the targeting guide RNA sequence (if applicable) and how the editor was applied.*
Authentication	*Describe any authentication procedures for each seed stock used or novel genotype generated. Describe any experiments used to assess the effect of a mutation and, where applicable, how potential secondary effects (e.g. second site T-DNA insertions, mosiacism, off-target gene editing) were examined.*
